# A Unifying Generator Loss Function for Generative Adversarial Networks

**DOI:** 10.3390/e26040290

**Published:** 2024-03-27

**Authors:** Justin Veiner, Fady Alajaji, Bahman Gharesifard

**Affiliations:** 1 Department of Mathematics and Statistics, Queen’s University, Kingston, ON K7L 3N6, Canada; justin.veiner@queensu.ca; 2 Department of Electrical and Computer Engineering, University of California, Los Angeles, CA 90095, USA; gharesifard@ucla.edu

**Keywords:** generative adversarial networks, deep learning, parameterized loss functions, *f*-divergence, Jensen-*f*-divergence

## Abstract

A unifying α-parametrized generator loss function is introduced for a dual-objective generative adversarial network (GAN) that uses a canonical (or classical) discriminator loss function such as the one in the original GAN (VanillaGAN) system. The generator loss function is based on a symmetric class probability estimation type function, $L_{\alpha }$, and the resulting GAN system is termed $L_{\alpha }$-GAN. Under an optimal discriminator, it is shown that the generator’s optimization problem consists of minimizing a Jensen-$f_{\alpha }$-divergence, a natural generalization of the Jensen-Shannon divergence, where $f_{\alpha }$ is a convex function expressed in terms of the loss function $L_{\alpha }$. It is also demonstrated that this $L_{\alpha }$-GAN problem recovers as special cases a number of GAN problems in the literature, including VanillaGAN, least squares GAN (LSGAN), least *k*th-order GAN (L*k*GAN), and the recently introduced $\left(\alpha_{D},\alpha_{G}\right)$-GAN with $\alpha_{D}=1$. Finally, experimental results are provided for three datasets—MNIST, CIFAR-10, and Stacked MNIST—to illustrate the performance of various examples of the $L_{\alpha }$-GAN system.

## 1. Introduction

Generative adversarial networks (GANs), first introduced by Goodfellow et al. in 2014 [1], have a variety of applications in media generation [2], image restoration [3], and data privacy [4]. GANs aim to generate synthetic data that closely resemble the original real data with (unknown) underlying distribution $P_{x}$. The GAN is trained such that the distribution of the generated data, $P_{g}$, approximates $P_{x}$ well. More specifically, low-dimensional random noise is fed to a generator neural network *G* to produce synthetic data. Real data and the generated data are then given to a discriminator neural network *D* that scores the data between 0 and 1, with a score close to 1 meaning that the discriminator thinks the data belong to the real dataset. The discriminator and generator play a minimax game, where the aim is to minimize the generator’s loss and maximize the discriminator’s loss.

Since its initial introduction, several variants of GAN have been proposed. Deep convolutional GAN (DCGAN) [5] utilizes the same loss functions as VanillaGAN (the original GAN) while combining GANs with convolutional neural networks, which are helpful when applying GANs to image data as they extract visual features from the data. DCGANs are more stable than the baseline model but can suffer from mode collapse, which occurs when the generator learns that a select number of images can easily fool the discriminator, resulting in the generator only generating those images. Another notable issue with VanillaGAN is the tendency for the generator network’s gradients to vanish. In the early stages of training, the discriminator lacks confidence and assigns generated data values close to zero. Therefore, the objective function tends to zero, resulting in small gradients and a lack of learning. To mitigate this issue, a non-saturating generator loss function was proposed in [1] so that gradients do not vanish early on in training.

In the original (VanillaGAN) problem setup, the objective function, expressed as a negative sum of two Shannon cross-entropies, is to be minimized by the generator and maximized by the discriminator. It is demonstrated that if the discriminator is fixed to be optimal (i.e., as a maximizer of the objective function), the GAN’s minimax game can be reduced to minimizing the Jensen-Shannon divergence (JSD) between the real and generated data’s probability distributions [1]. An analogous result was proven in [6] for RényiGANs, a dual-objective GAN using distinct discriminator and generator loss functions. More specifically, under a canonical discriminator loss function (as in [1]) and a generator loss function expressed in terms of two Rényi cross-entropies, it is shown that the RényiGAN optimization problem reduces to minimizing the Jensen-Rényi divergence, hence extending VanillaGAN’s results.

Nowozin et al. generalized VanillaGAN by formulating a class of loss functions in [7] parametrized by a lower semicontinuous convex function *f*, devising *f*-GAN. More specifically, the *f*-GAN problem consists of minimizing an *f*-divergence between the true data distribution and the generator distribution via a minimax optimization of a Fenchel conjugate representation of the *f*-divergence, where the VanillaGAN discriminator’s role (as a binary classifier) is replaced by a variational function estimating the ratio of the true data and generator distributions. The *f*-GAN loss function may be tedious to derive, as it requires computation of the Fenchel conjugate of *f*. It can be shown that *f*-GAN can interpolate between VanillaGAN and HellingerGAN, among others [7].

More recently, α-GAN was presented in [8], for which the aim is to derive a class of loss functions parameterized by α>0 and expressed in terms of a class probability estimation (CPE) loss between a real label y∈{0,1} and predicted label $\hat{y}\in \left[0,1\right]$ [8]. The ability to control α as a hyperparameter is beneficial to be able to apply one system to multiple datasets, as two datasets may be optimal under different α values. This work was further analyzed in [9] and expanded in [10] by introducing the dual-objective $\left(\alpha_{D},\alpha_{G}\right)$-GAN, which allowed for the generator and discriminator loss functions to have distinct α parameters with the aim of improving training stability. When $\alpha_{D}=\alpha_{G}$, the α-GAN optimization reduces to minimizing an Arimoto divergence, as originally derived in [8]. Note that α-GAN can recover several *f*-GANs, such as HellingerGAN, VanillaGAN, WassersteinGAN, and total variation GAN [8]. Furthermore, in their more recent work [11] that unifies [8,9,10], the authors establish, under some conditions, a one-to-one correspondence between CPE-loss-based GANs (such as α-GANs) and *f*-GANs that use a symmetric *f*-divergence (see Theorems 4–5 and Corollary 1 in [11]). They also prove various generalization and estimation error bounds for $\left(\alpha_{D},\alpha_{G}\right)$-GANs and illustrate their ability to mitigate training instability for synthetic Gaussian data as well as the Celeb-A and LSUN Classroom image datasets. The various $\left(\alpha_{D},\alpha_{G}\right)$-GAN equilibrium results do not provide an analogous result to JSD and Jensen-Rényi divergence minimization for the VanillaGAN [1] and RényiGAN [6] problems, respectively, as they do not involve a *Jensen-type divergence*. More specifically given a divergence measure D(p∥q) between distributions *p* and *q* (i.e., a positive-definite bivariate function: D(p∥q)≥0 with equality if and only if (iff) p=q almost everywhere (a.e.)), a *Jensen-type divergence of D* is given by

$$\frac{1}{2}D\left(p∥\frac{p+q}{2}\right)+\frac{1}{2}D\left(q∥\frac{p+q}{2}\right);$$

i.e., it is the arithmetic average of two D-divergences: one between *p* and the mixture (p+q)/2 and the other between *q* and (p+q)/2.

The main objective of our work is to present a unifying approach that provides an axiomatic framework to encompass several existing GAN generator loss functions so that GAN optimization can be simplified in terms of a Jensen-type divergence. In particular, our framework classifies the set of α-parameterized CPE-based loss functions $L_{\alpha }$, generalizing the α-loss function in [8,9,10,11]. We then propose $L_{\alpha }$-GAN: a dual-objective GAN that uses a function from this class for the generator and uses any canonical discriminator loss function that admits the same optimizer as VanillaGAN [1]. We show that under some regularity (convexity/concavity) conditions on $L_{\alpha }$, the minimax game played with these two loss functions is equivalent to the minimization of a Jensen-$f_{\alpha }$-divergence: a Jensen-type divergence and another natural extension of the Jensen-Shannon divergence (in addition to the Jensen-Rényi divergence [6]), where the generating function $f_{\alpha }$ of the divergence is directly computed from the CPE loss function $L_{\alpha }$. This result recovers various prior dual-objective GAN equilibrium results, thus unifying them under one parameterized generator loss function. The newly obtained Jensen-$f_{\alpha }$-divergence, which is noted to belong to the class of symmetric *f*-divergences with different generating functions (see Remark 1), is a useful measure of dissimilarity between distributions as it requires a convex function *f* with a restricted domain given by the interval [0,2] (see Remark 2) in addition to its symmetry and finiteness properties.

The rest of the paper is organized as follows. In Section 2, we review *f*-divergence measures and introduce the Jensen-*f*-divergence as an extension of the Jensen-Shannon divergence. In Section 3, we establish our main result regarding the optimization of our unifying generator loss function (Theorem 1) and show that it can be applied to a large class of known GANs (Lemmas 2–4). We conduct experiments in Section 4 by implementing different manifestations of $L_{\alpha }$-GAN on three datasets: MNIST, CIFAR-10, and Stacked MNIST. Finally, we conclude the paper in Section 5.

## 2. Preliminaries

We begin by presenting key information measures used throughout the paper. Let f:[0,∞)→(−∞,∞] be a convex continuous function that is strictly convex at 1 (i.e., $f\left(\lambda u_{1}+\left(1-\lambda \right)u_{2}\right)<\lambda f\left(u_{1}\right)+\left(1-\lambda \right)f\left(u_{2}\right)$ for all $u_{1},u_{2}\geq 0$, $u_{1}\neq u_{2}$, and λ∈(0,1) such that $\lambda u_{1}+\left(1-\lambda \right)u_{2}=1$) and satisfying

f(1)=0.

Note that the convexity of *f* already implies its continuity on (0,∞). Here, the continuity of *f* at 0 is extended, setting $f\left(0\right)=\lim_{u↓0}f\left(u\right)$, which may be infinite. Otherwise, f(u) is assumed to be finite for u>0.

**Definition** **1**  ([12,13,14])**.** *The **f-divergence** between two probability densities p and q with common support* $R\subseteq R^{d}$ *on the Lebesgue measurable space* (R,B(R),μ) *is denoted by* $D_{f}\left(p∥q\right)$ *and given by* 

(1) $$\begin{array}{c} D_{f}(p∥q)=\int_{R}q\;f\left(\frac{p}{q}\right)\;d\mu , \end{array}$$

*where we have used the shorthand* $\int_{R}g\;d\mu :=\int_{R}g\left(x\right)\;d\mu \left(x\right)$*, where g is a measurable function; we follow this convention from now on. Here, f is referred to as the generating function of* $D_{f}\left(p∥q\right)$.

For simplicity, we consider throughout densities with common supports. A comprehensive definition of *f*-divergence for arbitrary distributions can be found in Section III of [15]. We require that *f* is strictly convex around 1 and that it satisfies the normalization condition f(1)=0 to ensure positive-definiteness of the *f*-divergence, i.e., $D_{f}\left(p∥q\right)\geq 0$ with equality holding iff p=q (a.e.). We present examples of *f*-divergences under various choices of their generating function *f* in Table 1. We will be invoking these divergence measures in different parts of the paper.

The Rényi divergence of order α (α>0, α≠1) between densities *p* and *q* with common support R is used in [6] in the RényiGAN problem; it is given by [23,24]

(2) $$\begin{array}{c} D_{\alpha }(p∥q)=\frac{1}{\alpha -1}\log\left(\int_{R}p^{\alpha }q^{1-\alpha }\;d\mu \right). \end{array}$$

Note that the Rényi divergence is not an *f*-divergence; however, it can be expressed as a transformation of the Hellinger divergence (which is itself an *f*-divergence):

(3) $$\begin{array}{c} D_{\alpha }(p∥q)=\frac{1}{\alpha -1}\log\left(1+\left(\alpha -1\right)H_{\alpha }\left(p∥q\right)\right). \end{array}$$

We now introduce a new measure, the Jensen-*f*-divergence, which is analogous to the Jensen-Shannon and Jensen-Rényi divergences.

**Definition** **2.**  *The **Jensen-f-divergence** between two probability distributions p and q with common support $R\subseteq R^{d}$ on the Lebesgue measurable space (R,B(R),μ) is denoted by ${\mathrm{JD}}_{f}\left(p∥q\right)$ and given by* 

(4) $$\begin{array}{c} {\mathrm{JD}}_{f}(p∥q)=\frac{1}{2}D_{f}\left(p||\frac{p+q}{2}\right)+\frac{1}{2}D_{f}\left(q||\frac{p+q}{2}\right), \end{array}$$

 *where $D_{f}\left(\cdot ∥\cdot \right)$ is the f-divergence.*

We next verify that the Jensen-Shannon divergence is a Jensen-*f*-divergence.

**Lemma** **1.**  *Let p and q be two densities with common support $R\subseteq R^{d}$, and consider the function f:[0,∞)→(−∞,∞] given by f(u)=ulogu. Then we have that*

(5) $$\begin{array}{c} {\mathrm{JD}}_{f}(p∥q)=\mathrm{JSD}(p∥q). \end{array}$$

**Proof.**  As *f* is convex (and continuous) on its domain with f(1)=0, we have that

$$\begin{array}{cc} \mathrm{JSD}(p∥q) & =\frac{1}{2}\mathrm{KL}\left(p||\frac{p+q}{2}\right)+\frac{1}{2}\mathrm{KL}\left(q||\frac{p+q}{2}\right)\\ \; & =\frac{1}{2}\int_{R}p\log\left(\frac{2p}{p+q}\right)\;d\mu +\frac{1}{2}\int_{R}q\log\left(\frac{2q}{p+q}\right)\;d\mu \\ \; & =\frac{1}{2}\int_{R}\frac{p+q}{2}\left(\frac{2p}{p+q}\log\left(\frac{2p}{p+q}\right)\right)\;d\mu \\ \; & \;+\frac{1}{2}\int_{R}\frac{p+q}{2}\left(\frac{2q}{p+q}\log\left(\frac{2q}{p+q}\right)\right)\;d\mu \\ \; & ={\mathrm{JD}}_{f}\left(p∥q\right). \end{array}$$

□

**Remark** **1**  (*Jensen-f-divergence is a symmetric f-divergence*)**.** *Note that ${\mathrm{JD}}_{f}\left(p∥q\right)$ is itself a symmetric f-divergence (with a modified generating function). Indeed, given the continuous convex function f that is strictly convex around* 1 *with f(1)=0, consider the functions*

$$f_{1}(u):=\frac{u+1}{2}\;f\left(\frac{2u}{u+1}\right),\;u\geq 0,$$

 *and*

$$f_{2}(u):=\frac{u+1}{2}\;f\left(\frac{2}{u+1}\right),\;u\geq 0,$$

*which are both continuous convex, strictly convex around* 1, *and satisfy $f_{1}\left(1\right)=f_{2}\left(1\right)=0$. Now, direct calculations yield that*

$$D_{f}\left(p||\frac{p+q}{2}\right)=D_{f_{1}}\left(p∥q\right)$$

 *and*

$$D_{f}\left(q||\frac{p+q}{2}\right)=D_{f_{2}}\left(p∥q\right).$$

*Thus,*

$$\begin{array}{cc} {\mathrm{JD}}_{f}\left(p∥q\right) & =\frac{1}{2}D_{f_{1}}\left(p∥q\right)+\frac{1}{2}D_{f_{2}}\left(p∥q\right)=D_{\bar{f}}\left(p∥q\right), \end{array}$$

 *where $\bar{f}:=\frac{1}{2}\left(f_{1}+f_{2}\right)$, i.e.,*

(6) $$\bar{f}\left(u\right)=\frac{u+1}{4}\left(f\left(\frac{2u}{u+1}\right)+f\left(\frac{2}{u+1}\right)\right),\;u\geq 0,$$

*is also continuous convex, strictly convex around* 1*, and satisfies* $\bar{f}\left(1\right)=0$*. Since by* (4),

$${\mathrm{JD}}_{f}\left(p∥q\right)={\mathrm{JD}}_{f}\left(q∥p\right),$$

*we conclude that the Jensen-f-divergence is a symmetric $\bar{f}$-divergence. An equivalent argument is to note that $\bar{f}={\bar{f}}^{★}$, where ${\bar{f}}^{★}(u):=u\bar{f}\left(\frac{1}{u}\right)$, u≥0 (with ${\bar{f}}^{★}\left(0\right)=\lim_{t\to \infty }\bar{f}\left(t\right)/t$), which is a necessary and sufficient condition for the $\bar{f}$-divergence to be symmetric (see p. 4399 in [15]).*

**Remark** **2**  (*Domain of f*)**.** *Examining (4), we note that the Jensen-f-divergence between p and q involves the f-divergences between either p or q and their mixture (p+q)/2. In other words, to determine ${\mathrm{JD}}_{f}\left(p∥q\right)$, we only need $f\left(\frac{2p}{p+q}\right)$ and $f\left(\frac{2q}{p+q}\right)$ when taking the expectations in (1). Thus, it is sufficient to restrict the domain of the convex function f to the interval* [0,2].

## 3. Main Results

We now present our main theorem that unifies various generator loss functions under a CPE-based loss function $L_{\alpha }$ for a dual-objective GAN, $L_{\alpha }$-GAN, with a canonical discriminator loss function that is optimized as in [1]. Under some regularity conditions on the loss function $L_{\alpha }$, we show that under the optimal discriminator, our generator loss becomes a Jensen-*f*-divergence.

Let (X,B(X),μ) be the measured space of n×n×m images (where m=1 for black and white images and m=3 for RGB images), and let (Z,B(Z),μ) be a measured space such that $Z\subseteq R^{d}$. The discriminator neural network is given by D:X→[0,1], and the generator neural network is given by G:Z→X. The generator’s noise input is sampled from a multivariate Gaussian distribution $P_{z}:Z\to \left[0,1\right]$. We denote the probability distribution of the real data by $P_{x}:X\to \left[0,1\right]$ and the probability distribution of the generated data by $P_{g}:X\to \left[0,1\right]$. We also set $P_{x}$ and $P_{g}$ as the densities corresponding to $P_{x}$ and $P_{g}$, respectively. We begin by introducing the $L_{\alpha }$-GAN system.

**Definition** **3.**  *Fix α∈A⊆R and let $L_{\alpha }:\left\{0,1\right\}\times \left[0,1\right]\to \left[0,\infty \right)$ be a loss function such that $\hat{y}L_{\alpha }\left(1,\frac{\hat{y}}{2}\right)$ is a continuous function that is either convex or concave in $\hat{y}\in \left[0,2\right]$ with strict convexity (respectively, strict concavity) around $\hat{y}=1$ and such that $L_{\alpha }$ is symmetric in the sense that*

(7) $$\begin{array}{c} L_{\alpha }(1,\hat{y})=L_{\alpha }\left(0,1-\hat{y}\right),\;\hat{y}\in \left[0,1\right]. \end{array}$$

*Then the* $L_{\alpha }$**-GAN** *system is defined by $\left(V_{D},V_{L_{\alpha },G}\right)$, where $V_{D}:X\times Z\to R$ is the discriminator loss function, and $V_{L_{\alpha },G}:X\times Z\to R$ is the generator loss function, which is given by*

(8) $$\begin{array}{cc} V_{L_{\alpha },G}\left(D,G\right) & =E_{A∼P_{x}}\left[-L_{\alpha }\left(1,D\left(A\right)\right)\right]+E_{B∼P_{g}}\left[-L_{\alpha }\left(0,D\left(B\right)\right)\right]. \end{array}$$

*Moreover, the* $L_{\alpha }$**-GAN** ***problem** is defined by*

(9) $$\begin{array}{cc} \; & \underset{D}{\sup}\;V_{D}\left(D,G\right) \end{array}$$

(10) $$\begin{array}{cc} \; & \underset{G}{\inf}\;V_{L_{\alpha },G}\left(D,G\right). \end{array}$$

We now present our main result about the $L_{\alpha }$-GAN optimization problem.

**Theorem** **1.**  *For a fixed α∈A⊆R and $L_{\alpha }:\left\{0,1\right\}\times \left[0,1\right]\to \left[0,\infty \right)$, let $\left(V_{D},V_{L_{\alpha },G}\right)$ be the loss functions of $L_{\alpha }$-GAN and consider joint optimization in (9)–(10). If $V_{D}$ is a canonical loss function in the sense that it is maximized at $D=D^{*}$, where*

(11) $$\begin{array}{c} D^{*}=\frac{P_{x}}{P_{x}+P_{g}}, \end{array}$$

 *then (10) reduces to*

(12) $$\begin{array}{c} \underset{G}{\inf}V_{L_{\alpha },G}(D^{*},G)=\underset{G}{\inf}2a{\mathrm{JD}}_{f\alpha }\left(P_{x}∥P_{g}\right)-2ab, \end{array}$$

*where ${\mathrm{JD}}_{f\alpha }\left(\cdot ∥\cdot \right)$ is the Jensen-$f_{\alpha }$-divergence, and $f_{\alpha }:\left[0,2\right]\to R$ is a continuous convex function that is strictly convex around* 1 *and is given by*

(13) $$\begin{array}{c} f_{\alpha }(u)=-u\left(\frac{1}{a}L_{\alpha }\left(1,\frac{u}{2}\right)-b\right), \end{array}$$

*where a and b are real constants chosen so that $f_{\alpha }\left(1\right)=0$ with a<0 (respectively, a>0) if $uL_{\alpha }\left(1,\frac{u}{2}\right)$ is convex (respectively, concave). Finally, (12) is minimized when $P_{x}=P_{g}$ (a.e.).*

**Proof.**  Under the assumption that $V_{D}$ is maximized at $D^{*}=\frac{P_{x}}{P_{x}+P_{g}}$, we have that

$$\begin{array}{cc} V_{L_{\alpha },G}\left(D^{*},G\right) & =E_{A∼P_{x}}\left[-L_{\alpha }\left(1,D^{*}\left(A\right)\right)\right]+E_{B∼P_{g}}\left[-L_{\alpha }\left(0,D^{*}\left(B\right)\right)\right]\\ \; & =-\int_{X}P_{x}L_{\alpha }\left(1,D^{*}\right)\;d\mu -\int_{X}P_{g}L_{\alpha }\left(0,D^{*}\right)\;d\mu \\ \; & =-\int_{X}P_{x}L_{\alpha }\left(1,\frac{P_{x}}{P_{x}+P_{g}}\right)\;d\mu -\int_{X}P_{g}L_{\alpha }\left(0,\frac{P_{x}}{P_{x}+P_{g}}\right)\;d\mu \\ \; & =-2\int_{X}\left(\frac{P_{x}+P_{g}}{2}\right)\frac{P_{x}}{P_{x}+P_{g}}L_{\alpha }\left(1,\frac{P_{x}}{P_{x}+P_{g}}\right)\;d\mu \\ & \;-2\int_{X}\left(\frac{P_{x}+P_{g}}{2}\right)\frac{P_{g}}{P_{x}+P_{g}}L_{\alpha }\left(0,\frac{P_{x}}{P_{x}+P_{g}}\right)\;d\mu \\ \; & \overset{\left(a\right)}{=}-2\int_{X}\left(\frac{P_{x}+P_{g}}{2}\right)\frac{P_{x}}{P_{x}+P_{g}}L_{\alpha }\left(1,\frac{P_{x}}{P_{x}+P_{g}}\right)\;d\mu \\ & \;-2\int_{X}\left(\frac{P_{x}+P_{g}}{2}\right)\frac{P_{g}}{P_{x}+P_{g}}L_{\alpha }\left(1,\frac{P_{g}}{P_{x}+P_{g}}\right)\;d\mu \\ \; & \overset{\left(b\right)}{=}-2\int_{X}\left(\frac{P_{x}+P_{g}}{2}\right)\frac{P_{x}}{P_{x}+P_{g}}\left(\frac{-af_{\alpha }\left(\frac{2P_{x}}{P_{x}+P_{g}}\right)}{\frac{2P_{x}}{P_{x}+P_{g}}}+ab\right)\;d\mu \\ \; & \;-2\int_{X}\left(\frac{P_{x}+P_{g}}{2}\right)\frac{P_{g}}{P_{x}+P_{g}}\left(\frac{-af_{\alpha }\left(\frac{2P_{g}}{P_{x}+P_{g}}\right)}{\frac{2P_{g}}{P_{x}+P_{g}}}+ab\right)\;d\mu \\ \; & =2a\left(\frac{1}{2}\int_{X}\frac{P_{x}+P_{g}}{2}f_{\alpha }\left(\frac{2P_{x}}{P_{x}+P_{g}}\right)\;d\mu \right.\\ \; & \;+\left.\frac{1}{2}\int_{X}\frac{P_{x}+P_{g}}{2}f_{\alpha }\left(\frac{2P_{g}}{P_{x}+P_{g}}\right)\;d\mu \right)-2ab\\ \; & =2a\;{\mathrm{JD}}_{f\alpha }\left(P_{x}∥P_{g}\right)-2ab, \end{array}$$

where:

- (a) holds since $L_{\alpha }\left(1,u\right)=L_{\alpha }\left(0,1-u\right)$ by (7), where $u=\frac{P_{x}}{P_{x}+P_{g}}$.
- (b) holds by solving for $L_{\alpha }\left(1,u\right)$ in terms of $f_{\alpha }\left(2u\right)$ in (13), where $u=\frac{P_{x}}{P_{x}+P_{g}}$ in the first term and $u=\frac{P_{g}}{P_{x}+P_{g}}$ in the second term.

The constants *a* and *b* are chosen so that $f_{\alpha }\left(1\right)=0$. Finally, the continuity and convexity of $f_{\alpha }$ (as well as its strict convexity around 1) directly follow from the corresponding assumptions imposed on the loss function $L_{\alpha }$ in Definition 3 and on the condition imposed on the sign of *a* in the theorem’s statement.    □

**Remark** **3.**  *Note that not only $D^{*}$ given in (11) is an optimal discriminator of the (original) VanillaGAN discriminator loss function, but it also optimizes the LSGAN/LkGAN discriminators loss functions when their discriminators’ labels for fake and real data, γ and β, respectively satisfy γ=1 and β=0 (see Section 3.3).*

We next show that the $L_{\alpha }$-GAN of Theorem 1 recovers as special cases a number of well-known GAN generator loss functions and their equilibrium points (under an optimal classical discriminator $D^{*}$).

### 3.1. VanillaGAN

VanillaGAN [1] uses the same loss function $V_{\mathrm{VG}}$ for the both generator and discriminator, which is

(14) $$\begin{array}{c} V_{\mathrm{VG}}(D,G)=E_{A∼P_{x}}\left[-\log D\left(A\right)\right]+E_{B∼P_{g}}\left[-\log\left(1-D\left(B\right)\right)\right] \end{array}$$

and can be cast as a saddle point optimization problem:

(15) $$\begin{array}{c} \underset{G}{\inf}\underset{D}{\sup}V_{VG}\left(D,G\right). \end{array}$$

It is shown in [1] that the optimal discriminator for (15) is given by $D^{*}=\frac{P_{x}}{P_{x}+P_{g}}$, as in (11). When $D=D^{*}$, the optimization reduces to minimizing the Jensen-Shannon divergence:

(16) $$\begin{array}{c} \underset{G}{\inf}\;V_{\mathrm{VG}}(D^{*},G)=\underset{G}{\inf}\;2\mathrm{JSD}\left(P_{x}∥P_{g}\right)-2\log2. \end{array}$$

We next show that (16) can be obtained from Theorem 1.

**Lemma** **2.**  *Consider the optimization of VanillaGAN given in (15). Then we have that* 

$$\begin{array}{c} V_{\mathrm{VG}}(D^{*},G)=2\mathrm{JSD}\left(P_{x}∥P_{g}\right)-2\log2=V_{L_{\alpha },G}\left(D^{*},G\right), \end{array}$$

*where* $L_{\alpha }\left(y,\hat{y}\right)=-y\log\left(\hat{y}\right)-\left(1-y\right)\log\left(1-\hat{y}\right)$ *for all* α∈A=R.

**Proof.**  For any fixed α∈R, let the function $L_{\alpha }$ in (8) be as defined in the statement:

$$\begin{array}{c} L_{\alpha }(y,\hat{y})=-y\log\left(\hat{y}\right)-\left(1-y\right)\log\left(1-\hat{y}\right). \end{array}$$

Note that $L_{\alpha }$ is symmetric, since for $\hat{y}\in \left[0,1\right]$, we have that

$$\begin{array}{c} L_{\alpha }\left(1,\hat{y}\right)=-\log\left(\hat{y}\right)=L_{\alpha }\left(0,1-\hat{y}\right). \end{array}$$

Instead of showing the continuity and convexity/concavity conditions imposed on $\hat{y}L_{\alpha }\left(1,\frac{\hat{y}}{2}\right)$ in Definition 3, we implicitly verify them by directly deriving $f_{\alpha }$ from $L_{\alpha }$ using (13) and showing that it is continuous convex and strictly convex around 1. Setting a=1 and b=log2, we have that

$$\begin{array}{cc} f_{\alpha }\left(u\right) & =-u\left(\frac{1}{a}L_{\alpha }\left(1,\frac{u}{2}\right)-b\right)\\ \; & =-u\left(-\log\frac{u}{2}-\log2\right)=u\log u. \end{array}$$

Clearly, *f* is convex (actually strictly convex on (0,∞) and hence strictly convex around 1) and continuous on its domain (where $f\left(0\right)=\lim_{u\to 0}u\log\left(u\right)=0$). It also satisfies f(1)=0. By Lemma 1, we know that under the generating function f(u)=ulog(u), the Jensen-*f* divergence reduces to the Jensen-Shannon divergence. Therefore, by Theorem 1, we have that

$$\begin{array}{cc} V_{L_{\alpha },G}\left(D^{*},G\right) & =2a{\mathrm{JD}}_{f\alpha }\left(P_{x}∥P_{g}\right)-2ab\\ \; & =2\mathrm{JSD}(P_{x}∥P_{g})-2\log2\\ \; & =V_{\mathrm{VG}}\left(D^{*},G\right), \end{array}$$

which finishes the proof.    □

### 3.2. α-GAN

The notion of α-GANs is introduced in [8] as a way to unify several existing GANs using a parameterized loss function. We describe α-GANs next.

**Definition** **4**  ([8])**.** *Let y∈{0,1} be a binary label, $\hat{y}\in \left[0,1\right]$, and fix α>0. The **α-loss** between y and $\hat{y}$ is the map $\ell_{\alpha }:\left\{0,1\right\}\times \left[0,1\right]\to \left[0,\infty \right)$ given by*

(17) $$\ell_{\alpha }\left(y,\hat{y}\right)=\{\begin{array}{cc} \frac{\alpha }{\alpha -1}\left(1-y{\hat{y}}^{\frac{\alpha -1}{\alpha }}+\left(1-y\right){\left(1-\hat{y}\right)}^{\frac{\alpha -1}{\alpha }}\right), & \alpha \in (0,1)\cup (1,\infty )\\ -y\log\hat{y}-\left(1-y\right)\log\left(1-\hat{y}\right), & \alpha =1. \end{array}$$

**Definition** **5**  ([8])**.** *For α>0, the α***-GAN *loss function*** *is given by*

(18) $$\begin{array}{c} V_{\alpha }(D,G)=E_{A∼P_{x}}\left[-\ell_{\alpha }\left(1,D\left(A\right)\right)\right]+E_{B∼P_{g}}\left[-\ell_{\alpha }\left(0,D\left(B\right)\right)\right]. \end{array}$$

*Joint optimization of the α-GAN problem is given by*

(19) $$\begin{array}{c} \underset{G}{\inf}\;\underset{D}{\sup}\;V_{\alpha }\left(D,G\right). \end{array}$$

It is known that α-GAN recovers several well-known GANs by varying the α parameter: notably, VanillaGAN (α=1) [1] and HellingerGAN ($\alpha =\frac{1}{2}$) [7]. Furthermore, as α→∞, $V_{\alpha }$ recovers a translated version of the WassersteinGAN loss function [25]. We now present the solution to the joint optimization problem presented in (19).

**Proposition** **1**  ([8])**.** *Let α>0 and consider joint optimization of the α-GAN presented in (19). The discriminator $D^{*}$ that maximizes the loss function is given by*

(20) $$\begin{array}{cc} D^{*} & =\frac{{P_{x}}^{\alpha }}{{P_{x}}^{\alpha }+{P_{g}}^{\alpha }}. \end{array}$$

*Furthermore, when $D=D^{*}$ is fixed, the problem in (19) reduces to minimizing an Arimoto divergence (as defined in Table 1) when α≠1:*

(21) $$\begin{array}{c} \underset{G}{\inf}V_{\alpha }(D^{*},G)=\underset{G}{\inf}A_{\alpha }\left(P_{x}∥P_{g}\right)+\frac{\alpha }{\alpha -1}\left(2^{\frac{1}{\alpha }}-2\right) \end{array}$$

 *and a Jensen-Shannon divergence when α=1:*

(22) $$\begin{array}{c} \underset{G}{\inf}V_{1}(D^{*},G)=\underset{G}{\inf}\mathrm{JSD}\left(P_{x}∥P_{g}\right)-2\log2, \end{array}$$

 *where (21) and (22) achieve their minima iff $P_{x}=P_{g}$ (a.e.).*

Recently, α-GAN was generalized in [10] to implement a dual-objective GAN, which we describe next.

**Definition** **6**  ([10])**.** *For $\alpha_{D}>0$ and $\alpha_{G}>0$, the $\left(\alpha_{D},\alpha_{G}\right)$***-GAN’*****s optimization** is given by*

(23) $$\begin{array}{cc} \; & \underset{D}{\sup}\;V_{\alpha_{D}}\left(D,G\right) \end{array}$$

(24) $$\begin{array}{cc} \; & \underset{G}{\inf}\;V_{\alpha_{G}}\left(D,G\right) \end{array}$$

*where $V_{\alpha_{D}}$ and $V_{\alpha_{G}}$ are defined in (18), with α replaced by $\alpha_{D}$ and $\alpha_{G}$, respectively.*

**Proposition** **2**  ([10])**.** *Consider the joint optimization in (23) and (24). Let parameters $\alpha_{D}$, $\alpha_{G}>0$ satisfy*

(25) $$\begin{array}{c} \left(\alpha_{D}\leq 1,\alpha_{G}>\frac{\alpha_{D}}{\alpha_{D}+1}\right)\;\mathrm{or}\;\left(\alpha_{D}>1,\frac{\alpha_{D}}{2}<\alpha_{G}\leq \alpha_{D}\right). \end{array}$$

*The discriminator $D^{*}$ that maximizes $V_{\alpha_{D}}$ is given by*

(26) $$\begin{array}{cc} D^{*} & =\frac{{P_{x}}^{\alpha_{D}}}{{P_{x}}^{\alpha_{D}}+{P_{g}}^{\alpha_{D}}}. \end{array}$$

*Furthermore, when $D=D^{*}$ is fixed, the minimization of $V_{\alpha_{G}}$ in (24) is equivalent to the following f-divergence minimization:*

(27) $$\begin{array}{c} \underset{G}{\inf}\;V_{\alpha_{G}}(D^{*},G)=\underset{G}{\inf}\;D_{f_{\alpha_{D},\alpha_{G}}}\left(P_{x}∥P_{g}\right)+\frac{\alpha }{\alpha -1}\left(2^{\frac{1}{\alpha }}-2\right), \end{array}$$

 *where $f_{\alpha_{D},\alpha_{G}}:\left[0,\infty \right)\to R$ is given by*

(28) $$\begin{array}{cc} f_{\alpha_{D},\alpha_{G}}\left(u\right) & =\frac{\alpha_{G}}{\alpha_{G}-1}\left(\frac{u^{\alpha_{D}\left(1-\frac{1}{\alpha_{G}}\right)+1}+1}{{\left(u^{\alpha_{D}}+1\right)}^{1-\frac{1}{\alpha_{G}}}}\right). \end{array}$$

We now apply the $\left(\alpha_{D},\alpha_{G}\right)$-GAN to our main result in Theorem 1 by showing that (12) can recover (27) when $\alpha_{D}=1$ (which corresponds to a VanillaGAN discriminator loss function).

**Lemma** **3.** *Consider the $\left(\alpha_{D},\alpha_{G}\right)$-GAN given in Definition 6. Let $\alpha_{D}=1$ and $\alpha_{G}=\alpha >\frac{1}{2}$. Then, the solution to (24) presented in Proposition 2 is equivalent to minimizing a Jensen-$f_{\alpha }$-divergence: specifically, if $D^{*}$ is the optimal discriminator given by (26), which is equivalent to (11) when $\alpha_{D}=1$, then $V_{\alpha ,G}\left(D^{*},G\right)$ in (27) satisfies*

(29) $$\begin{array}{cc} V_{\alpha ,G}\left(D^{*},G\right) & =2^{\frac{1}{\alpha }}{\mathrm{JD}}_{f\alpha }\left(P_{x}∥P_{g}\right)+\frac{\alpha }{\alpha -1}\left(2^{\frac{1}{\alpha }}-2\right)=V_{L_{\alpha },G}\left(D^{*},G\right), \end{array}$$

 *where $L_{\alpha }\left(y,\hat{y}\right)=\ell_{\alpha }\left(y,\hat{y}\right)$, and*

(30) $$\begin{array}{c} f_{\alpha }\left(u\right)=\frac{\alpha }{\alpha -1}\left(u^{2-\frac{1}{\alpha }}-u\right),\;u\geq 0. \end{array}$$

**Proof.**  We show that Theorem 1 recovers Proposition 2 by setting $L_{\alpha }\left(y,\hat{y}\right)=\ell_{\alpha }\left(y,\hat{y}\right)$. Note that $\ell_{\alpha }$ is symmetric since

$$\begin{array}{c} \ell_{\alpha }\left(1,\hat{y}\right)=\frac{\alpha }{\alpha -1}\left(1-{\hat{y}}^{1-\frac{1}{\alpha }}\right)=\ell_{\alpha }\left(0,1-\hat{y}\right). \end{array}$$

As in the proof of Lemma 2, instead of proving the conditions imposed on $\hat{y}L_{\alpha }\left(1,\frac{\hat{y}}{2}\right)$ in Definition 3, we derive $f_{\alpha }$ directly from $L_{\alpha }$ using (13) and show that it is continuous convex and strictly convex around 1. From Lemma 2, we know that when α=1, $f_{\alpha }\left(u\right)=u\log u$ (which is strictly convex and continuous). For α∈(0,1)∪(1,∞), setting $a=2^{\frac{1}{\alpha }-1}$ and $b=\frac{\alpha }{\alpha -1}\left(2^{1-\frac{1}{\alpha }}-1\right)$ in (13), we have that

$$\begin{array}{cc} f_{\alpha }\left(u\right) & =-u\left(\frac{1}{a}L_{\alpha }\left(1,\frac{u}{2}\right)-b\right)\\ \; & =-u\left(2^{1-\frac{1}{\alpha }}\frac{\alpha }{\alpha -1}\left(1-{\left(\frac{u}{2}\right)}^{1-\frac{1}{\alpha }}\right)-\frac{\alpha }{\alpha -1}\left(2^{1-\frac{1}{\alpha }}-1\right)\right)\\ \; & =\frac{\alpha }{\alpha -1}\left(-u\right)\left[2^{1-\frac{1}{\alpha }}-u^{1-\frac{1}{\alpha }}-\left(2^{1-\frac{1}{\alpha }}-1\right)\right]\\ \; & =\frac{\alpha }{\alpha -1}\left(u^{2-\frac{1}{\alpha }}-u\right). \end{array}$$

Clearly, $f_{\alpha }\left(1\right)=0$. Furthermore for α≠1, we have that

$$\begin{array}{cc} f_{\alpha }^{\prime \prime }\left(u\right) & =\frac{\left(2\alpha -1\right)u^{\frac{-1}{\alpha }}}{\alpha },\;u\geq 0, \end{array}$$

which is positive for $\alpha >\frac{1}{2}$, and $f_{\alpha }$ is convex for $\alpha >\frac{1}{2}$ (as well as continuous on its domain and strictly convex around 1). Thus, by Theorem 1, we have that

$$\begin{array}{cc} V_{L_{\alpha },G}\left(D^{*},G\right) & =2a{\mathrm{JD}}_{f\alpha }\left(P_{x}∥P_{g}\right)-2ab\\ \; & =2\;\cdot \;2^{\frac{1}{\alpha }-1}{\mathrm{JD}}_{f\alpha }\left(P_{x}∥P_{g}\right)-2\frac{\alpha }{\alpha -1}2^{\frac{1}{\alpha }-1}\left(2^{1-\frac{1}{\alpha }}-1\right)\\ \; & =2^{\frac{1}{\alpha }}{\mathrm{JD}}_{f\alpha }\left(P_{x}∥P_{g}\right)+\frac{\alpha }{\alpha -1}\left(2^{\frac{1}{\alpha }}-2\right). \end{array}$$

We now show that the above Jensen-$f_{\alpha }$-divergence is equal to the $f_{1,\alpha }$-divergence originally derived for the (1,α)-GAN problem of Proposition 2 (note from Proposition 2 that if $\alpha_{D}=1$, then $\alpha_{G}=\alpha >\frac{1}{2}$, so the range of α concurs with the range required above for the convexity of $f_{\alpha }$). For any two distributions *p* and *q* with common support X, we have that

$$\begin{array}{cc} D_{f_{1,\alpha }}\left(p∥q\right) & =\frac{\alpha }{\alpha -1}\int_{X}q\frac{{\left(\frac{p}{q}\right)}^{2-\frac{1}{\alpha }}+1}{{\left(\frac{p}{q}+1\right)}^{1-\frac{1}{\alpha }}}\;d\mu -\frac{\alpha }{\alpha -1}2^{\frac{1}{\alpha }}\\ \; & =\frac{\alpha }{\alpha -1}\int_{X}q\frac{{\left(\frac{p}{q}\right)}^{2-\frac{1}{\alpha }}+1}{{\left(\frac{p+q}{q}\right)}^{1-\frac{1}{\alpha }}}\;d\mu -\frac{\alpha }{\alpha -1}2^{\frac{1}{\alpha }}\\ \; & =\frac{\alpha }{\alpha -1}\int_{X}\left(\left(p+q\right){\left(\frac{p}{p+q}\right)}^{2-\frac{1}{\alpha }}+\left(p+q\right){\left(\frac{q}{p+q}\right)}^{2-\frac{1}{\alpha }}\right)\;d\mu \\ & \;-\frac{\alpha }{\alpha -1}2^{\frac{1}{\alpha }}\\ \; & =\frac{\alpha }{\alpha -1}\frac{2}{2^{2-\frac{1}{\alpha }}}\int_{X}\left(\frac{p+q}{2}{\left(\frac{2p}{p+q}\right)}^{2-\frac{1}{\alpha }}+\frac{p+q}{2}{\left(\frac{2q}{p+q}\right)}^{2-\frac{1}{\alpha }}\right)\;d\mu \\ & \;-\frac{\alpha }{\alpha -1}2^{\frac{1}{\alpha }}\\ \; & =\frac{\alpha }{\alpha -1}2^{\frac{1}{\alpha }-1}\int_{X}\left(\frac{p+q}{2}\left({\left(\frac{2p}{p+q}\right)}^{2-\frac{1}{\alpha }}-\frac{2p}{p+q}\right)+p\right)\;d\mu \\ \; & \;+\frac{\alpha }{\alpha -1}2^{\frac{1}{\alpha }-1}\int_{X}\left(\frac{p+q}{2}\left({\left(\frac{2q}{p+q}\right)}^{2-\frac{1}{\alpha }}-\frac{2q}{p+q}\right)+q\right)\;d\mu \\ & \;\;-\frac{\alpha }{\alpha -1}2^{\frac{1}{\alpha }}\\ \; & =\frac{\alpha }{\alpha -1}2^{\frac{1}{\alpha }}\frac{1}{2}\left(\int_{X}\frac{p+q}{2}\left({\left(\frac{2p}{p+q}\right)}^{2-\frac{1}{\alpha }}-\frac{2p}{p+q}\right)\;d\mu +1\right)\\ \; & \;+\frac{\alpha }{\alpha -1}2^{\frac{1}{\alpha }}\frac{1}{2}\left(\int_{X}\frac{p+q}{2}\left({\left(\frac{2q}{p+q}\right)}^{2-\frac{1}{\alpha }}-\frac{2q}{p+q}\right)\;d\mu +1\right)\\ & \;\;-\frac{\alpha }{\alpha -1}2^{\frac{1}{\alpha }}\\ \; & =2^{\frac{1}{\alpha }}{\mathrm{JD}}_{f\alpha }\left(p∥q\right)+\frac{\alpha }{\alpha -1}2^{\frac{1}{\alpha }-1}\left(2\right)-\frac{\alpha }{\alpha -1}2^{\frac{1}{\alpha }}\\ \; & =2^{\frac{1}{\alpha }}{\mathrm{JD}}_{f\alpha }\left(p∥q\right). \end{array}$$

Therefore, $V_{L_{\alpha },G}\left(D^{*},G\right)=V_{\alpha }\left(D^{*},G\right)$.    □

Note that this lemma generalizes Lemma 2; VanillaGAN is a special case of (1,α)-GAN for α=1.

### 3.3. Shifted LkGANs and LSGANs

Least squares GAN (LSGAN) was proposed in [26] to mitigate the vanishing gradient problem with VanillaGAN and to stabilize training performance. LSGAN’s loss function is derived from the squared error distortion measure, whereby we aim to minimize the distortion between the data samples and a target value we want the discriminator to assign the samples to. LSGAN was generalized with L*k*GAN in [6] by replacing the squared error distortion measure with an absolute error distortion measure of order k≥1, therefore introducing an additional degree of freedom to the generator’s loss function. We first state the general L*k*GAN problem. We then apply the result of Theorem 1 to the loss functions of LSGAN and L*k*GAN.

**Definition** **7**  ([6])**.** *Let γ, β,*c∈[0,1] *, and let *k≥1. ***LkGAN’s loss functions**, denoted by* $V_{\mathrm{LSGAN},D}$ *and* $V_{k,G}$*, are given by*

(31) $$\begin{array}{cc} V_{\mathrm{LSGAN},D}\left(D,G\right) & =-\frac{1}{2}E_{A∼P_{x}}\left[{\left(D\left(A\right)-\beta \right)}^{2}\right]-\frac{1}{2}E_{B∼P_{g}}\left[{\left(D\left(B\right)-\gamma \right)}^{2}\right] \end{array}$$

(32) $$\begin{array}{cc} V_{k,G}\left(D,G\right) & =E_{A∼P_{x}}{\left[|D\left(A\right)-c\right|}^{k}]+E_{B∼P_{g}}{\left[|D\left(B\right)-c\right|}^{k}]. \end{array}$$

*The **LkGAN problem** is the joint optimization*

(33) $$\begin{array}{cc} \; & \underset{D}{\sup}\;V_{\mathrm{LSGAN},D}\left(D,G\right) \end{array}$$

(34) $$\begin{array}{cc} \; & \underset{G}{\inf}\;V_{k,G}\left(D,G\right). \end{array}$$

We next recall the solution to (33), which is a minimization of the Pearson–Vajda divergence ${\left|\chi \right|}^{k}\left(\cdot ∥\cdot \right)$ of order *k* (as defined in Table 1).

**Proposition** **3**  ([6])**.** *Consider the joint optimization for LkGAN presented in (33). Then the optimal discriminator $D^{*}$ that maximizes $V_{\mathrm{LSGAN},D}$ in (31) is given by*

(35) $$\begin{array}{cc} D^{*} & =\frac{\gamma P_{x}+\beta P_{g}}{P_{x}+P_{g}}. \end{array}$$

*Furthermore, if $D=D^{*}$ and γ−β=2(c−β), the minimization of $V_{k,G}$ in (32) reduces to*

(36) $$\begin{array}{cc} \underset{G}{\inf}\;V_{k,G}\left(D,G\right) & =\underset{G}{\inf}{\;|c-\beta |}^{k}{\left|\chi \right|}^{k}\left(P_{x}+P_{g}∥2P_{g}\right). \end{array}$$

Note that LSGAN [26] is a special case of L*k*GAN, as we recover LSGAN when k=2 [6].

By scrutinizing Proposition 3 and Theorem 1, we observe that the former cannot be recovered from the latter. However, we can use Theorem 1 by slightly modifying the L*k*GAN generator’s loss function. First, for the dual-objective GAN proposed in Theorem 1, we need $D^{*}=\frac{P_{x}}{P_{x}+P_{g}}$. By (35), this is achieved for γ=1 and β=0. Then, we define the intermediate loss function

(37) $$\begin{array}{cc} {\tilde{V}}_{k,G}\left(D,G\right) & =E_{A∼P_{x}}[|D\left(A\right)-c_{1}{|}^{k}]+E_{B∼P_{g}}[|D\left(B\right)-c_{2}{|}^{k}]. \end{array}$$

Comparing the above loss function with (8), we note that setting $c_{1}=0$ and $c_{2}=1$ in (37) satisfies the symmetry property of $L_{\alpha }$. Finally, to ensure the generating function $f_{\alpha }$ satisfies $f_{\alpha }\left(1\right)=0$, we shift each term in (37) by 1. Putting these changes together, we propose a revised generator loss function denoted by ${\hat{V}}_{k,G}$ and given by

(38) $$\begin{array}{c} {\hat{V}}_{k,G}\left(D,G\right)=E_{A∼P_{x}}{\left[|D\left(A\right)\right|}^{k}-1]+E_{B∼P_{g}}{\left[|1-D\left(B\right)\right|}^{k}-1]. \end{array}$$

We call a system that uses (38) as a generator loss function a **Shifted LkGAN (SLkGAN)**. If k=2, we have a shifted version of the LSGAN generator loss function, which we call **Shifted LSGAN (SLSGAN)**. Note that none of these modifications alter the gradients of $V_{k,G}$ in (32), since the first term is independent of *G*, the choice of $c_{1}$ is irrelevant, and translating a function by a constant does not change its gradients. However, from Proposition 3, for γ=0, β=1, and c=1, we do not have that γ−β=2(c−β), and as a result, this modified problem does not reduce to minimizing a Pearson–Vajda divergence. Consequently, we can relax the condition on *k* in Definition 7 to just k>0. We now show how Theorem 1 can be applied to $L_{\alpha }$-GAN using (38).

**Lemma** **4.**  *Let k>0. Let $V_{D}$ be a discriminator loss function, and let ${\hat{V}}_{k,G}$ be the generator’s loss function defined in (38). Consider the joint optimization*

(39) $$\begin{array}{cc} \; & \underset{D}{\sup}V_{D}\left(D,G\right) \end{array}$$

(40) $$\begin{array}{cc} \; & \underset{G}{\inf}{\hat{V}}_{k,G}\left(D,G\right) \end{array}$$

*If $V_{D}$ is optimized at $D^{*}=\frac{P_{x}}{P_{x}+P_{g}}$ (i.e., $V_{D}$ is canonical), then we have that*

$$\begin{array}{c} {\hat{V}}_{k,G}(D^{*},G)=\frac{1}{2^{k-1}}{\mathrm{JD}}_{fk}\left(P_{x}∥P_{g}\right)+\frac{1}{2^{k-1}}-\frac{1}{2}, \end{array}$$

 *where $f_{k}$ is given by*

$$\begin{array}{c} f_{k}(u)=u(u^{k}-1),\;u\geq 0. \end{array}$$

Examples of $V_{D}\left(D,G\right)$ that satisfy the requirements of Lemma 4 include the L*k*GAN discriminator loss function given by (31) with γ=1 and β=0 and the VanillaGAN discriminator loss function given by (14).

**Proof.**  Let k>0. We can restate SL*k*GAN’s generator loss function in (38) in terms of $V_{L_{\alpha },G}$ in (8): we have that $V_{L_{\alpha },G}\left(D^{*},G\right)={\hat{V}}_{k,G}\left(D^{*},G\right)$, where α=k, and $L_{k}:\left\{0,1\right\}\times \left[0,1\right]\to \left[0,\infty \right)$ is given by

(41) $$\begin{array}{c} L_{k}(y,\hat{y})=-(y\left({\hat{y}}^{k}-1\right)+\left(1-y\right)\left({\left(1-\hat{y}\right)}^{k}-1\right)). \end{array}$$

We have that $L_{k}$ is symmetric, since

$$\begin{array}{cc} L_{k}\left(1,\hat{y}\right) & =-\left({\hat{y}}^{k}-1\right)=L_{k}\left(0,1-\hat{y}\right). \end{array}$$

We derive $f_{\alpha }$ from $L_{\alpha }$ via (13) and directly check that it is continuous convex and strictly convex around 1. Setting $a=\frac{1}{2^{k}}$ and $b=2^{k}-1$ in (13), we have that

$$\begin{array}{cc} f_{k}\left(u\right) & =-u\left(\frac{1}{a}L_{k}\left(1,\frac{u}{2}\right)-b\right)\\ \; & =-u\left(2^{k}\left(1-{\left(\frac{u}{2}\right)}^{k}\right)-\left(2^{k}-1\right)\right)\\ \; & =-u(2^{k}-u^{k}-2^{k}+1)\\ \; & =u(u^{k}-1). \end{array}$$

We clearly have that $f_{k}\left(1\right)=0$ and that $f_{k}$ is continuous. Furthermore, we have that $f_{k}^{\prime \prime }\left(u\right)=k\left(k+1\right)u$, which is non-negative for u≥0. Therefore, $f_{k}$ is convex (as well as strictly convex around 1). As a result, by Theorem 1, we have that

$$\begin{array}{cc} {\hat{V}}_{k,G}\left(D^{*},G\right) & =\frac{1}{2^{k-1}}{\mathrm{JD}}_{fk}\left(P_{x}∥P_{g}\right)-\frac{1}{2^{k-1}}\left(2^{k}-1\right)\\ \; & =\frac{1}{2^{k-1}}{\mathrm{JD}}_{fk}\left(P_{x}∥P_{g}\right)+\frac{1}{2^{k-1}}-\frac{1}{2}. \end{array}$$

□

We conclude this section by emphasizing that Theorem 1 serves as a unifying result recovering the existing loss functions in the literature and, moreover, provides a way for generalizing new ones. Our aim in the next section is to demonstrate the versatility of this result in experimentation.

## 4. Experiments

We perform two experiments on three different image datasets that we describe below.

**Experiment 1**: In the first experiment, we compare (α,α)-GAN with (1,α)-GAN while controlling the value of α. Recall that $\alpha_{D}=1$ corresponds to the canonical VanillaGAN (or DCGAN) discriminator. We aim to verify whether or not replacing an α-GAN discriminator with a VanillaGAN discriminator stabilizes or improves the system’s performance depending on the value of α. Note that the result of Theorem 1 only applies to the $\left(\alpha_{D},\alpha_{G}\right)$-GAN for $\alpha_{D}=1$. We herein confine the comparison of (1,α)-GAN with (α,α)-GAN only so that both systems have the same tunable free parameter α. The results obtained in [10] for the Stacked MNIST dataset show that $\left(\alpha_{D},\alpha_{G}\right)$-GAN provides consistently robust performance when $\alpha_{D}=\alpha_{G}$. Other experiments illustrating the performance of $\left(\alpha_{D},\alpha_{G}\right)$-GAN with $\alpha_{D}\neq 1$ are carried for the Celeb-A and LSUN Classroom image datasets in [11] and show improved training stability for $\alpha_{D}<1$ values.

**Experiment 2**: We train two variants of SL*k*GAN with the generator loss function as described in (38) and parameterized by k>0. We then utilize two different canonical discriminator loss functions to align with Theorem 1. The first is the VanillaGAN discriminator loss given by (14); we call the resulting dual-objective GAN **Vanilla-SLkGAN**. The second is the L*k*GAN discriminator loss given by (31), where we set γ=1 and β=0 such that the optimal discriminator is given by (11). We call this system **Lk-SLkGAN**. We compare the two variants to analyze how the value of *k* and choice of discriminator loss impacts the system’s performance.

### 4.1. Experimental Setup

We run both experiments on three image datasets: MNIST [27], CIFAR-10 [28], and Stacked MNIST [29]. The MNIST dataset is a dataset of black and white handwritten digits between 0 and 9 and with a size of 28×28×1. The CIFAR-10 dataset is an RGB dataset of small images of common animals and modes of transportation with a size of 32×32×3. The Stacked MNIST dataset is an RGB dataset derived from the MNIST dataset and constructed by taking three MNIST images, assigning each to one of the three color channels, and stacking the images on top of each other. The resulting images are then padded so that each one of them has a size of 32×32×3.

For Experiment 1, we use α values of 0.5, 5.0, 10.0, and 20.0. For each value of α, we train (α, α)-GAN and (1,α)-GAN. We additionally train DCGAN, which corresponds to (1,1)-GAN. For Experiment 2, we use *k* values of 0.25, 1.0, 2.0, 7.5, and 15.0. Note that when k=2, we recover LSGAN. For the MNIST dataset, we run 10 trials with the random seeds 123, 500, 1600, 199,621, 60,677, 20,435, 15,859, 33,764, 79,878, and 36,123 and train each GAN for 250 epochs. For the RGB datasets (CIFAR-10 and Stacked MNIST), we run five trials with the random seeds 123, 1600, 60,677, 15,859, and 79,878 and train each GAN for 500 epochs. All experiments utilize an Adam optimizer for the stochastic gradient descent algorithm with a learning rate of $2\times 10^{-4}$ and parameters $\beta_{1}=0.5$, $\beta_{2}=0.999$, and $\epsilon =10^{-7}$ [30]. We also experiment with the addition of a gradient penalty (GP); we add a penalty term to the discriminator’s loss function to encourage the discriminator’s gradient to have a unit norm [31].

The MNIST experiments were run on one 6130 2.1 GHz 1xV100 GPU, 8 CPUs, and 16 GB of memory. The CIFAR-10 and Stacked MNIST experiments were run on one Epyc 7443 2.8 GHz GPU, 8 CPUs, and 16 GB of memory. For each experiment, we report the best overall Fréchet inception distance (FID) score [32], the best average FID score amongst all trials and its variance, and the average epoch the best FID score occurs and its variance. The FID score for each epoch was computed over 10,000 images. For each metric, the lowest numerical value corresponds to the model with the best metric (indicated in bold in the tables). We also report how many trials we include in our summary statistics, as it is possible for a trial to collapse and not train for the full number of epochs. The neural network architectures used in our experiments are presented in Appendix A. The training algorithms are presented in Appendix B.

### 4.2. Experimental Results

**Figure 1:**
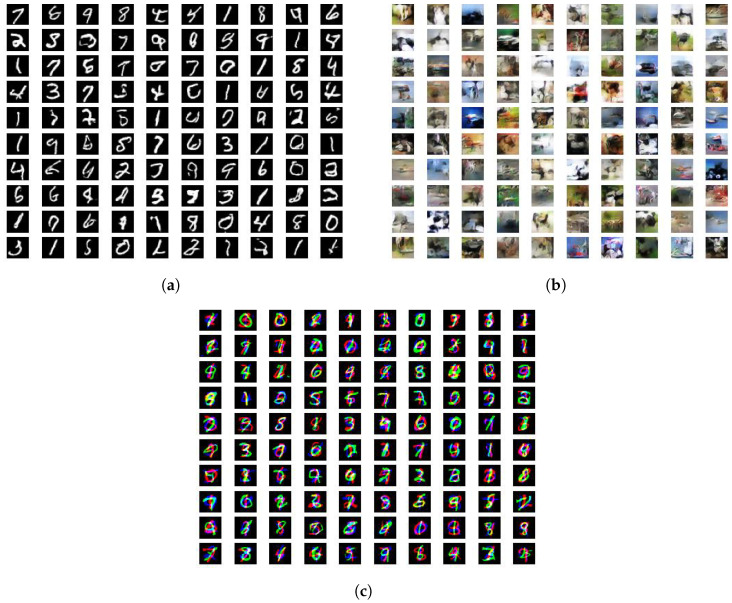
Generated images for the best-performing ($\alpha_{D}$, $\alpha_{G}$)-GANs. (**a**) ($\alpha_{D},\alpha_{G}$)-GAN for MNIST, $\alpha_{D}=1.0$, $\alpha_{G}=5.0$, FID: 1.125. (**b**) $\left(\alpha_{D},\alpha_{G}\right)$-GAN-GP for CIFAR-10, $\alpha_{D}=1.0$, $\alpha_{G}=20.0$, FID = 8.466. (**c**) $\left(\alpha_{D},\alpha_{G}\right)$-GAN-GP for Stacked MNIST, $\alpha_{D}=1.0$, $\alpha_{G}=0.5$, FID = 4.833.

**Figure 2:**
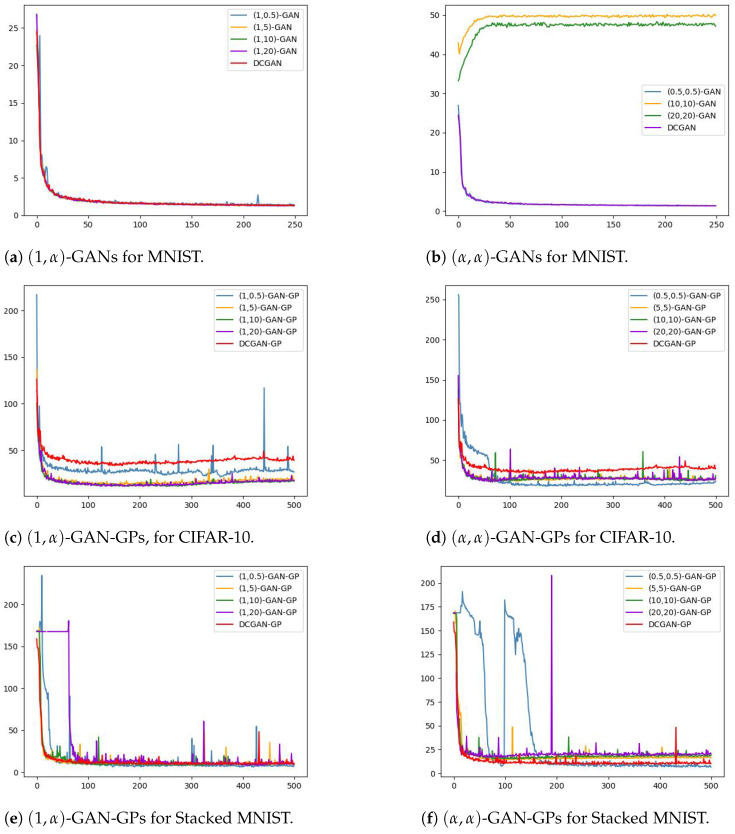
Average FID scores vs. epochs for various $\left(\alpha_{D},\alpha_{G}\right)$-GANs.

**Figure 3:**
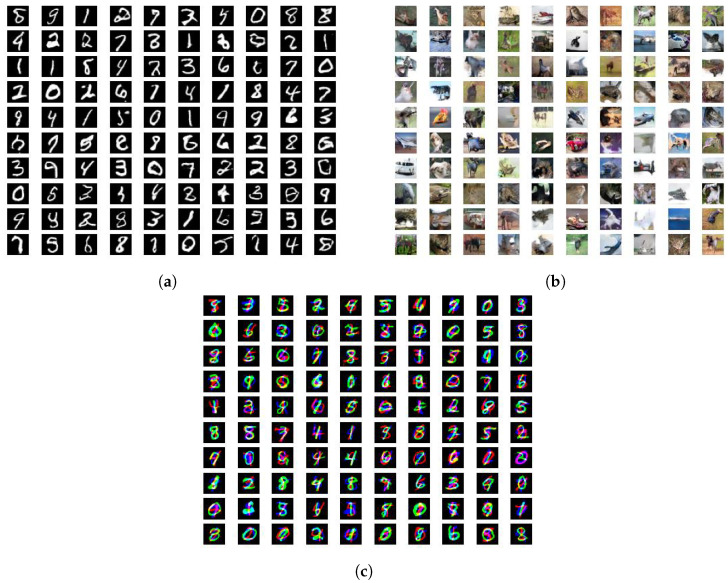
Generated images for best-performing SL*k*GANs. (**a**) Vanilla-SL*k*GAN-0.25 for MNIST, FID = 1.112. (**b**) Vanilla-SL*k*GAN-2.0 for CIFAR-10, FID = 4.58. (**c**) Vanilla-SL*k*GAN-15.0-GP for Stacked MNIST, FID = 3.836.

**Figure 4:**
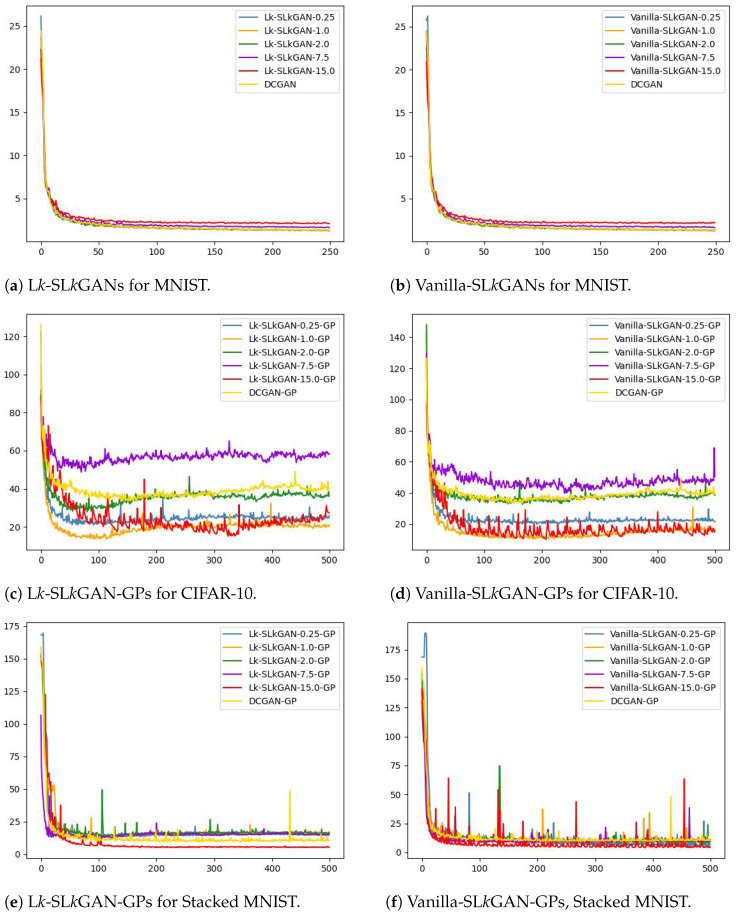
FID scores vs. epochs for various SL*k*GANs.

We report the FID metrics for Experiment 1 in Table 2, Table 3 and Table 4 and for Experiment 2 in Table 5, Table 6 and Table 7. We report only on those experiments that produced meaningful results. Models that utilize a simplified gradient penalty have the suffix “-GP”. For $\left(\alpha_{D},\alpha_{G}\right)$-GANs, we display the output of the best-performing systems in Figure 1 and plot the trajectories of the FID scores throughout the training epochs in Figure 2. Similarly for SL*K*GANs, outputs of the best-performing systems and FID scores vs. epochs trajectories are provided in Figure 3 and Figure 4, respectively.

### 4.3. Discussion

#### 4.3.1. Experiment 1

From Table 2, we note that 37 of the 90 trials collapse before 250 epochs have passed without a gradient penalty. The (5,5)-GAN collapses for all five trials, and hence, it is not displayed in Table 2. This behavior is expected, as (α,α)-GAN is more sensitive to exploding gradients when α does not tend to 0 or +∞ [8]. The addition of a gradient penalty could mitigate the discriminator’s gradients diverging in the (5,5)-GAN by encouraging gradients to have a unit norm. Using a VanillaGAN discriminator with an α-GAN generator (i.e., (1,α)-GAN) produces better quality images for all tested values of α compared to when both networks utilize an α-GAN loss function. The (1,10)-GAN achieves excellent stability, converging in all 10 trials, and also achieves the lowest average FID score. The (1,5)-GAN achieves the lowest FID score overall, marginally outperforming DCGAN. Note that when the average best FID score is very close to the best FID score, the resulting best FID score variance is quite small (of the order of $10^{-3}$), indicating little statistical variability over the trials.

Likewise, for the CIFAR-10 and Stacked MNIST datasets, (1,α)-GAN produces lower FID scores than (α,α)-GAN (see Table 3 and Table 4). However, both models are more stable with the CIFAR-10 dataset. With the exception of DCGAN, no model converged to its best FID score for all five trials with the Stacked MNIST dataset. Comparing the trials that did converge, both (α,α)-GAN and (1,α)-GAN performed better on the Stacked MNIST dataset than the CIFAR-10 dataset. For CIFAR-10, the (1,10)- and (1,20)-GANs produced the best overall FID score and the best average FID score, respectively. On the other hand, the (1,0.5)-GAN produced the best overall FID score and the best average FID score for the Stacked MNIST dataset. We also observe a tradeoff between speed and performance for the CIFAR-10 and Stacked MNIST datasets: the (1,α)-GANs arrive at their lowest FID scores later than their respective (α,α)-GANs but achieve lower FID scores overall.

Comparing Figure 2c and Figure 2d, we observe that (α,α)-GAN-GP provides more stability than (1,α)-GAN for lower values of α (i.e., α=0.5), while (1,α)-GAN-GP exhibits more stability for higher α values (α=10 and α=20). Figure 2e,f show that the two α-GANs trained on the Stacked MNIST dataset exhibit unstable behavior earlier into training when α=0.5 or α=20. However, both systems stabilize and converge to their lowest FID scores as training progresses. The (0.5,0.5)-GAN-GP system in particular exhibits wildly erratic behavior for the first 200 epochs then finishes training with a stable trajectory that outperforms DCGAN-GP.

A future direction is to explore how the complexity of an image dataset influences the best choice of α. For example, the Stacked MNIST dataset might be considered to be less complex than CIFAR-10, as images in the Stacked MNIST dataset only contain four unique colors (black, red, green, and blue), while the CIFAR-10 dataset utilizes significantly more colors.

#### 4.3.2. Experiment 2

We see from Table 5 that all L*k*-L*k*GANs and Vanilla-SL*k*GANs have FID scores comparable to the DCGAN. When k=15, Vanilla-SL*k*GAN and L*k*-SL*k*GAN arrive at their lowest FID scores slightly earlier than DCGAN and other SL*k*GANs.

The addition of a simplified gradient penalty is necessary for L*k*-SL*k*GAN to achieve overall good performance on the CIFAR-10 dataset (see Table 6). Interestingly, Vanilla-SL*k*GAN achieves lower FID scores without a gradient penalty for lower *k* values (k=1,2) and with a gradient penalty for higher *k* values (k=7.5,15). When k=0.25, both SL*k*GANs collapsed for all five trials without a gradient penalty.

Table 7 shows that Vanilla-SL*k*GANs achieve better FID scores than their respective L*k*-L*k*GAN counterparts. However, L*k*-L*k*GANs are more stable, as no single trial collapsed, while 10 of the 25 Vanilla-SL*k*GAN trials collapsed before 500 epochs had passed. While all Vanilla-SL*k*GANs outperform the DCGAN with a gradient penalty, L*k*-SL*k*GAN-GP only outperforms DCGAN-GP when k=15. Except for when k=7.5, we observe that the L*k*-SL*k*GAN system takes fewer epochs to arrive at its lowest FID score. Comparing Figure 4e and Figure 4f, we observe that L*k*-SL*k*GANs exhibit more stable FID score trajectories than their respective Vanilla-SL*k*GANs. This makes sense, as the L*k*GAN loss function aims to increase the GAN’s stability compared to DCGAN [6].

## 5. Conclusions

We introduced a parameterized CPE-based generator loss function for a dual-objective GAN termed $L_{\alpha }$-GAN that, when used in tandem with a canonical discriminator loss function that achieves its optimum in (11), minimizes a Jensen-$f_{\alpha }$-divergence. We showed that this system can recover VanillaGAN, (1,α)-GAN, and L*k*GAN as special cases. We conducted experiments with the three aforementioned $L_{\alpha }$-GANs on three image datasets. The experiments indicate that (1,α)-GAN exhibits better performance than (α,α)-GAN with α>1. They also show that the devised SL*k*GAN system achieves lower FID scores with a VanillaGAN discriminator compared with an L*k*GAN discriminator.

Future work consists of unveiling more examples of existing GANs that fall under our result as well as applying $L_{\alpha }$-GAN to novel, judiciously designed CPE losses $L_{\alpha }$ and evaluating the performance (in terms of both quality and diversity of generated samples) and the computational efficiency of the resulting models. Another interesting and related direction is to study $L_{\alpha }$-GAN within the context of *f*-GANs, given that the Jensen-*f*-divergence is itself an *f*-divergence (see Remark 1), by systematically analyzing different Jensen-*f*-divergences and the role they play in improving GAN performance and stability. Other worthwhile directions include incorporating the proposed $L_{\alpha }$ loss into state-of-the-art GAN models, such as, among others, BigGAN [33], StyleGAN [34], and CycleGAN [35], for high-resolution data generation and image-to-image translation applications and conducting a meticulous analysis of the sensitivity of the models’ performance to different values of the α parameter and providing guidelines on how best to tune α for different types of datasets.

## Figures and Tables

**Table 1:** Examples of *f*-divergences.

*f*-Divergence	Symbol	Formula	f(u)
Kullback–Leiber [16]	KL	$\int_{R}p\log\left(\frac{p}{q}\right)\;d\mu$	ulogu
Jensen-Shannon [17]	JSD	$\frac{1}{2}\mathrm{KL}\left(p||\frac{p+q}{2}\right)+\frac{1}{2}\mathrm{KL}\left(q||\frac{p+q}{2}\right)$	$\frac{1}{2}\left(u\log u-\left(u+1\right)\log\frac{u+1}{2}\right)$
Pearson $\chi^{2}$ [18]	$\chi^{2}$	$\int_{R}\frac{{\left(q-p\right)}^{2}}{p}\;d\mu$	${\left(\sqrt{x}-\frac{1}{\sqrt{x}}\right)}^{2}$
Pearson–Vajda (k>1) [18]	${\left|\chi \right|}^{k}$	$\int_{R}\frac{{\left|q-p\right|}^{k}}{p^{k-1}}\;d\mu$	$u^{1-k}{\left|1-u\right|}^{k}$
Arimoto (α>0, α≠1) [15,19,20]	$A_{\alpha }$	$\frac{\alpha }{\alpha -1}\left(\int_{R}{\left(p^{\alpha }+q^{\alpha }\right)}^{\frac{1}{\alpha }}\;d\mu -2^{\frac{1}{\alpha }}\right)$	$\frac{\alpha }{\alpha -1}\left({\left(1+u\right)}^{\frac{1}{\alpha }}-\left(1+u\right)-2^{\frac{1}{\alpha }}+2\right)$
Hellinger (α>0, α≠1) [15,21,22]	$H_{\alpha }$	$\frac{1}{\alpha -1}\left(\int_{R}p^{\alpha }q^{1-\alpha }\;d\mu -1\right)$	$\frac{u^{\alpha }-1}{\alpha -1}$

**Table 2:** $\left(\alpha_{D},\alpha_{G}\right)$-GAN results for MNIST.

($\alpha_{D},\alpha_{G}$)-GAN	Best FID Score	Average Best FID Score	Best FID Score Variance	Average Epochs	Epoch Variance	Number of Successful Trials (/10)
(1,0.5)-GAN	1.264	1.288	$2.979\times 10^{-4}$	227.25	420.25	4
(0.5,0.5)-GAN	1.209	1.265	0.001	234.5	156.7	6
(1,5)-GAN	1.125	1.17	$8.195\times 10^{-4}$	230.3	617.344	10
**(1,10)-GAN**	1.147	1.165	$7.984\times 10^{-4}$	225.6	253.156	10
(10,10)-GAN	36.506	39.361	16.312	1.5	0.5	2
(1,20)-GAN	1.135	1.174	0.001	237.5	274.278	10
(20,20)-GAN	33.23	33.23	0.0	1.0	0.0	1
DCGAN	1.154	1.208	0.001	231.3	357.122	10

**Table 3:** $\left(\alpha_{D},\alpha_{G}\right)$-GAN results for CIFAR-10.

($\alpha_{D},\alpha_{G}$)-GAN	Best FID Score	Average Best FID Score	Best FID Score Variance	Average Epochs	Epoch Variance	Number of Successful Trials (/5)
(1,0.5)-GAN-GP	10.551	14.938	12.272	326.2	1808.7	5
(0.5,0.5)-GAN-GP	13.734	14.93	0.517	223.6	11,378.3	5
(1,5)-GAN-GP	10.772	11.635	0.381	132.0	1233.5	5
(5,5)-GAN-GP	20.79	21.72	0.771	84.8	1527.2	5
**(1,10)-GAN-GP**	9.465	10.187	0.199	182.6	1096.3	5
(10,10)-GAN-GP	19.99	21.095	0.434	131.8	13,374.7	5
(1,20)-GAN-GP	8.466	10.217	1.479	216.2	6479.7	5
(20,20)-GAN-GP	19.378	21.216	2.315	138.2	29,824.2	5
DCGAN-GP	25.731	28.378	3.398	158.0	2510.5	5

**Table 4:** $\left(\alpha_{D},\alpha_{G}\right)$-GAN results for Stacked MNIST.

($\alpha_{D},\alpha_{G}$)-GAN	Best FID Score	Average Best FID Score	Best FID Score Variance	Average Epochs	Epoch Variance	Number of Successful Trials (/5)
**(1,0.5)-GAN-GP**	4.833	4.997	0.054	311.5	23,112.5	2
(0.5,0.5)-GAN-GP	6.418	6.418	0.0	479.0	0.0	1
(1,5)-GAN-GP	7.98	7.988	$1.357\times 10^{-4}$	379.5	11,704.5	2
(5,5)-GAN-GP	12.236	12.836	0.301	91.5	387.0	4
(1,10)-GAN-GP	7.502	7.528	0.001	326.5	14,280.5	2
(10,10)-GAN-GP	14.22	14.573	0.249	95.0	450.0	2
(1,20)-GAN-GP	8.379	8.379	0.0	427.0	0.0	1
(20,20)-GAN-GP	16.584	16.584	0.0	94.0	0.0	1
DCGAN-GP	7.507	7.774	0.064	303.4	11,870.8	5

**Table 5:** SL*k*GAN results for MNIST.

Variant-SL*k*GAN-*k*	Best FID Score	Average Best FID Score	Best FID Score Variance	Average Epochs	Epoch Variance	Number of Successful Trials (/10)
L*k*-SL*k*GAN-0.25	1.15	1.174	$6.298\times 10^{-4}$	224.3	940.9	10
**Vanilla-SLkGAN-0.25**	1.112	1.162	0.001	237.0	124.0	10
L*k*-SL*k*GAN-1.0	1.122	1.167	$8.857\times 10^{-4}$	233.0	124.0	10
Vanilla-SL*k*GAN-1.0	1.126	1.17	$9.218\times 10^{-4}$	226.2	1182.844	10
L*k*-SL*k*GAN-2.0	1.148	1.198	$5.248\times 10^{-4}$	237.2	288.4	10
Vanilla-SL*k*GAN-2.0	1.124	1.184	$8.933\times 10^{-4}$	237.8	138.4	10
L*k*-SL*k*GAN-7.5	1.455	1.498	$4.422\times 10^{-4}$	229.0	322.222	10
Vanilla-SL*k*GAN-7.5	1.439	1.511	0.001	212.2	1995.067	10
L*k*-SL*k*GAN-15.0	1.733	1.872	0.005	198.8	1885.733	10
Vanilla-SL*k*GAN-15.0	1.773	1.876	0.005	171.6	3122.267	10
DCGAN	1.154	1.208	0.001	231.3	357.122	10

**Table 6:** SL*k*GAN results for CIFAR-10.

Variant-SL*k*GAN-*k*	Best FID Score	Average Best FID Score	Best FID Score Variance	Average Epochs	Epoch Variance	Number of Successful Trials (/5)
L*k*-SL*k*GAN-1.0	4.727	118.242	10,914.643	60.8	1897.2	5
Vanilla-SL*k*GAN-1.0	4.821	5.159	0.092	88.0	506.5	5
L*k*-SL*k*GAN-2.0	4.723	145.565	7492.26	73.2	3904.2	5
**Vanilla-SLkGAN-2.0**	4.58	5.1	0.261	105.4	740.8	5
L*k*-SL*k*GAN-7.5	6.556	155.497	7116.521	254.6	18,605.3	5
Vanilla-SL*k*GAN-7.5	6.384	48.905	8698.195	72.2	1711.7	5
L*k*-SL*k*GAN-15.0	8.576	145.774	5945.097	263.0	36,463.0	5
Vanilla-SL*k*GAN-15.0	7.431	50.868	8753.002	82.6	3106.8	5
DCGAN	4.753	5.194	0.117	88.6	462.8	5
L*k*-SL*k*GAN-0.25-GP	17.366	18.974	2.627	87.8	1897.2	5
Vanilla-SL*k*GAN-0.25-GP	16.013	17.912	1.961	189.0	9487.5	5
L*k*-SL*k*GAN-1.0-GP	10.771	12.567	1.083	77.8	239.2	5
Vanilla-SL*k*GAN-1.0-GP	8.569	9.588	0.749	197.6	2690.3	5
L*k*-SL*k*GAN-2.0-GP	23.11	25.013	1.924	75.4	658.8	5
Vanilla-SL*k*GAN-2.0-GP	28.215	29.69	1.242	232.0	20,438.5	5
L*k*-SL*k*GAN-7.5-GP	33.304	41.48	49.187	82.8	1081.2	5
Vanilla-SL*k*GAN-7.5-GP	33.085	34.799	1.597	290.8	12,714.7	5
L*k*-SL*k*GAN-15.0-GP	9.157	12.504	3.839	310.4	6976.8	5
**Vanilla-SLkGAN-15.0-GP**	7.283	8.568	1.535	185.6	5978.3	5
DCGAN-GP	25.731	28.378	3.398	158.0	2510.5	5

**Table 7:** SL*k*GAN results for Stacked MNIST.

Variant-SL*k*GAN-*k*	Best FID Score	Average Best FID Score	Best FID Score Variance	Average Epochs	Epoch Variance	Number of Successful Trials (/5)
L*k*-SL*k*GAN-0.25-GP	10.541	11.824	0.678	113.6	356.3	5
Vanilla-SL*k*GAN-0.25-GP	5.197	5.197	0.0	496.0	0.0	1
L*k*-SL*k*GAN-1.0-GP	11.545	12.046	0.291	89.0	238.5	5
Vanilla-SL*k*GAN-1.0-GP	7.475	7.626	0.045	177.0	3528.0	2
L*k*-SL*k*GAN-2.0-GP	10.682	12.782	2.12	180.2	28,484.7	5
Vanilla-SL*k*GAN-2.0-GP	6.023	7.096	0.991	416.667	12,244.333	3
L*k*-SL*k*GAN-7.5-GP	8.912	9.906	0.577	239.0	35,663.5	5
Vanilla-SL*k*GAN-7.5-GP	6.074	6.43	0.164	238.0	21,729.5	5
L*k*-SL*k*GAN-15.0-GP	4.458	4.74	0.029	253.4	11,512.3	5
**Vanilla-SLkGAN-15.0-GP**	3.836	3.873	0.002	485.0	354.667	4
DCGAN-GP	7.507	7.774	0.064	303.4	11,870.8	5

## Data Availability

All codes used in our experiments can be found at this https://github.com/justin-veiner/MASc, accessed on 20 February 2024.

## Appendix A. Neural Network Architectures

We outline the architectures used for the generator and discriminator. For the MNIST dataset, we use the architectures of [6]. For the CIFAR-10 and Stacked MNIST datasets, we base the architectures on [5]. We summarize some aliases for the architectures in Table A1. For all models, we use a batch size of 100 and a noise size of 784 for the generator input.

**Table A1:** Summary of aliases used to describe neural network architectures.

Alias	Definition
FC	Fully Connected
UpConv2D	Deconvolutional Layer
Conv2D	Convolutional Layer
BN	Batch Normalization
LeakyReLU	Leaky Rectified Linear Unit

We omit the bias in the convolutional and deconvolutional layers to decrease the number of parameters being trained, which in turn decreases computation times. We initialize our kernels using a normal distribution with zero mean and variance 0.01. We present the MNIST architectures in Table A2 and Table A3 and the CIFAR-10 and Stacked MNIST architectures in Table A4 and Table A5.

**Table A2:** Discriminator architecture for the MNIST dataset.

Layer	Output Size	Kernel	Stride	BN	Activation
Input	28×28×1	No			
Conv2D	14×14×64	5×5	2	No	LeakyReLU (0.3)
Dropout (0.3)				No	
Conv2D	7×7×128	5×5	2	No	LeakyReLU (0.3)
Dropout(0.3)				No	
FC	1			No	Sigmoid

**Table A3:** Generator architecture for the MNIST dataset.

Layer	Output Size	Kernel	Stride	BN	Activation
Input	784				
FC	7×7×256				
UpConv2D	7×7×128	5×5	1	Yes	LeakyReLU (0.3)
UpConv2D	14×14×64	5×5	2	Yes	LeakyReLU (0.3)
UpConv2D	28×28×1	5×5	2	No	Tanh

**Table A4:** Discriminator architecture for the CIFAR-10 and Stacked MNIST datasets.

Layer	Output Size	Kernel	Stride	BN	Activation
Input	32×32×3				
Conv2D	16×16×128	3×3	2	No	LeakyReLU (0.2)
Conv2D	8×8×128	3×3	2	No	LeakyReLU (0.2)
Conv2D	4×4×256	3×3	2	No	LeakyReLU (0.2)
Dropout (0.4)				No	
FC	1				Sigmoid

**Table A5:** Generator architecture for the CIFAR-10 and Stacked MNIST datasets.

Layer	Output Size	Kernel	Stride	BN	Activation
Input	784				
FC	4×4×256				
UpConv2D	8×8×128	4×4	2	Yes	LeakyReLU (0.2)
UpConv2D	16×16×128	4×4	2	Yes	LeakyReLU (0.2)
UpConv2D	32×32×128	4×4	2	Yes	LeakyReLU (0.2)
Conv2D	32×32×3	3×3	1	No	Tanh

## Appendix B. Algorithms

We outline the algorithms used to train our models in Algorithms A1–A3.

**Algorithm A1** Overview of ($\alpha_{D}$, $\alpha_{G}$)-GAN training
**Require** $\alpha_{D}$, $\alpha_{G}$, number of epochs $n_{e}$, batch size *B*, learning rate η **Initialize** generator *G* with parameters $\theta_{G}$, discriminator *D* with parameters $\theta_{D}$. **for** i=1 to $n_{e}$ **do** **Sample** batch of real data $x=\{x_{1},\ldots ,x_{B}\}$ from dataset **Sample** batch of Gaussian noise vectors $z=\left\{z_{1},\ldots ,z_{B}\right\}∼N\left(0,I\right)$ **Update** the discriminator’s parameters using an Adam optimizer with learning rate η by descending the gradient: $$\begin{array}{c} \nabla_{\theta_{D}}\left(-\frac{1}{B}\sum_{i=1}^{B}\left(-\ell_{\alpha }\left(1,D\left(x_{i}\right)\right)-\ell_{\alpha }\left(0,D\left(G\left(z_{i}\right)\right)\right)\right)\right) \end{array}$$ or **update** the discriminator’s parameters with a simplified GP: $$\begin{array}{c} \nabla_{\theta_{D}}\left(-\frac{1}{B}\sum_{i=1}^{B}\left(-\ell_{\alpha }\left(1,D\left(x_{i}\right)\right)-\ell_{\alpha }\left(0,D\left(G\left(z_{i}\right)\right)\right)\right)\right.\;\\ \left.\;\;\;\;\;+5\left(\sum_{i=1}^{B}||\nabla_{x}\log\left(\frac{D(x)}{1-D(x)}\right)|{|}_{2}^{2}\right)\right) \end{array}$$ **Update** the generator’s parameters using an Adam optimizer with learning rate η and descending the gradient: $$\begin{array}{cc} \; & \nabla_{\theta_{G}}\left(\frac{1}{B}\sum_{i=1}^{B}\ell_{\alpha }\left(0,D\left(G\left(z_{i}\right)\right)\right)\right) \end{array}$$ **end for**

**Algorithm A2** Overview of L*k*-SL*k*GAN training
**Require** *k*, number of epochs $n_{e}$, batch size *B*, learning rate η **Initialize** generator *G* with parameters $\theta_{G}$, discriminator *D* with parameters $\theta_{D}$. **for** i=1 to $n_{e}$ **do** **Sample** batch of real data $x=\{x_{1},\ldots ,x_{B}\}$ from dataset **Sample** batch of Gaussian noise vectors $z=\left\{z_{1},\ldots ,z_{B}\right\}∼N\left(0,I\right)$ **Update** the discriminator’s parameters using an Adam optimizer with learning rate η by descending the gradient: $$\begin{array}{c} \nabla_{\theta_{D}}\left(\frac{1}{B}\sum_{i=1}^{B}\left(\frac{1}{2}{\left(D\left(x_{i}\right)-1\right)}^{2}+\frac{1}{2}\left(D{\left(G\left(z_{i}\right)\right)}^{2}\right)\right)\right) \end{array}$$ or **update** the discriminator’s parameters with a simplified GP: $$\begin{array}{c} \nabla_{\theta_{D}}\left(\frac{1}{B}\sum_{i=1}^{B}\left(\frac{1}{2}{\left(D\left(x_{i}\right)-1\right)}^{2}+\frac{1}{2}\left(D{\left(G\left(z_{i}\right)\right)}^{2}\right)\right)\right.\;\\ \;\;\;\;\;\left.+5\left(\sum_{i=1}^{B}||\nabla_{x}\log\left(\frac{D(x)}{1-D(x)}\right)|{|}_{2}^{2}\right)\right) \end{array}$$ **Update** the generator’s parameters using an Adam optimizer with learning rate η and descending the gradient: $$\begin{array}{cc} \; & \nabla_{\theta_{G}}\left(\frac{1}{B}\sum_{i=1}^{B}\frac{1}{2}(|1-D\left(G\left(z_{i}\right)\right){|}^{k}-1)\right) \end{array}$$ **end for**

**Algorithm A3** Overview of Vanilla-SL*k*GAN training
**Require** *k*, number of epochs $n_{e}$, batch size *B*, learning rate η **Initialize** generator *G* with parameters $\theta_{G}$, discriminator *D* with parameters $\theta_{D}$. **for** i=1 to $n_{e}$ **do** **Sample** batch of real data $x=\{x_{1},\ldots ,x_{B}\}$ from dataset **Sample** batch of noise vectors $z=\left\{z_{1},\ldots ,z_{B}\right\}∼N\left(0,I\right)$ **Update** the discriminator’s parameters using an Adam optimizer with learning rate η by descending the gradient: $$\begin{array}{c} \nabla_{\theta_{D}}\left(-\frac{1}{B}\sum_{i=1}^{B}\left(\log\left(D\left(x_{i}\right)\right)+\log\left(1-D\left(G\left(z_{i}\right)\right)\right)\right)\right) \end{array}$$ or **update** the discriminator’s parameters with a simplified (GP): $$\begin{array}{c} \nabla_{\theta_{D}}\left(-\frac{1}{B}\sum_{i=1}^{B}\left(\log\left(D\left(x_{i}\right)\right)+\log\left(1-D\left(G\left(z_{i}\right)\right)\right)\right)\right.\;\\ \;\;\;\;\;\left.+5\left(\sum_{i=1}^{B}||\nabla_{x}\log\left(\frac{D(x)}{1-D(x)}\right)|{|}_{2}^{2}\right)\right) \end{array}$$ **Update** the generator’s parameters using an Adam optimizer with learning rate η and descending the gradient: $$\begin{array}{c} \nabla_{\theta_{G}}\left(\frac{1}{B}\sum_{i=1}^{B}\frac{1}{2}(|1-D\left(G\left(z_{i}\right)\right){|}^{k}-1)\right) \end{array}$$ **end for**
